# Misinterpretation of Gram Stain from the Stationary Growth Phase of Positive Blood Cultures for *Brucella* and *Acinetobacter* Species

**DOI:** 10.2174/1874285801711010126

**Published:** 2017-06-30

**Authors:** Ali M. Bazzi, Jaffar A. Al-Tawfiq, Ali A. Rabaan

**Affiliations:** 1 *Microbiology Lab, Johns Hopkins Aramco Healthcare, Dhahran, Saudi Arabia*; 2 *Molecular Diagnostic Lab, Johns Hopkins Aramco Healthcare, Dhahran, Saudi Arabia*; 3 Specialty Internal Medicine, Johns Hopkins Aramco Healthcare, Dhahran, Saudi Arabia; 4 Department of Medicine Indiana University School of Medicine, Indianapolis, IN, USA

**Keywords:** Gram-stain, Blood culture, *Brucella*, *Acinetobacter*, *Coryneform*

## Abstract

**Introduction:**

*Acinetobacter baumannii* and *Brucella* species are Gram-negative organisms that are vulnerable to misinterpretation as Gram-positive or Gram-variable in blood cultures.

**Objective:**

We assess the random errors in gram stain interpretation to reduce the likelihood of such errors and therefore patient harm.

**Methodology:**

Aerobic and anaerobic blood cultures from two patients in an acute care facility in Saudi Arabia were subjected to preliminary Gram-staining. In case 1, VITEK-2 Anaerobe Identification, repeat Gram staining from a blood agar plate, Remel BactiDrop™ Oxidase test, Urea Agar urease test and real-time PCR were used to confirm presence of *Brucella* and absence of *Coryneform* species. In case 2, repeat Gram- staining from the plate and the vials, VITEK-2 Gram-Negative Identification, real-time PCR and subculture on to Columbia agar, blood agar, and MacConkey agar were carried out to identify *A. baumannii*.

**Results:**

In case 1, initially pleomorphic Gram-positive bacteria were identified. *Coryneform* species were suspected. Tiny growth was observed after 24 h on blood agar plates, and good growth by 48 h. Presence of *Brucella* species was ultimately confirmed. In case 2, preliminary Gram-stain results suggested giant Gram-positive oval cocci. Further testing over 18-24 h identified *A. baumannii*.

**Conclusions:**

Oxidase test from the plate and urease test from the culture vial is recommended after apparent identification of pleomorphic Gram-positive bacilli from blood culture, once tiny growth is observed, to distinguish *Brucella* from *Corynebacterium* species. If giant Gram-positive oval cocci are indicated by preliminary Gram-staining, it is recommended that the Gram stain be repeated from the plate after 4-6 h, or culture should be tested in Triple Sugar Iron (TSI) medium and the Gram stain repeated after 2-4 h incubation.

## INTRODUCTION

Misinterpretation of initial Gram-negative staining from clinical sample cultures can result in misidentification of the causative bacteria and delays appropriate antibiotic treatment. *Acinetobacter baumannii* are Gram-negative, strictly aerobic, catalase-positive, oxidase-negative bacteria which, in log phase, have the appearance of short, Gram-negative rods, but in stationary phase the organism has a coccoid appearance [1]. They are difficult to destain, which can lead to initial misidentification as Gram-positive or Gram-variable [1-3]. *A. baumannii* developed extensive resistance to many antibiotics, including broad-spectrum β-lactams and carbapenems, so rapid identification of the bacteria is imperative in making decisions on appropriate early treatment [2]. In Saudi Arabia, data on antibiotic susceptibility of *A. baumannii* is limited and suggest emergence of multi-drug resistant (MDR), nosocomial *A. baumannii* [4-9]. Other Gram-negative bacteria which are vulnerable to misidentification are *Brucella* species [10]. Brucellosis is rare in developed countries, however this zoonotic disease is of special concern in countries where there is significant importation of livestock, such as Saudi Arabia [11, 12]. It is also one of the common laboratory-acquired infections worldwide [10]. Primary Gram stain reporting as Gram-positive or Gram-variable can lead to misidentification of these slow-growing Gram-negative coccobacilli and a misidentification of contaminating *Coryneform* species.

Here we report two cases from an acute-care facility in which initial misinterpretation of Gram staining in blood cultures led to delays in identification of *Brucella* and *A. baumannii,* respectively.

## CASE REPORTS

### Case Report 1

In February 2015, a 12-year-old boy presented to the Emergency Room with fever, vomiting and diarrhea. The patient was given IV fluids, tests were performed and he was sent home. Blood culture turned out to be positive for gram positive cocci in pairs and chains and the patient was called back for admission. The patient prior to these symptoms went to the desert for the school vacations, where he had contact with animals. On admission, he was given IV ceftriaxone; later on, doxycycline and rifampin were added to his treatment. The patient improved his fever subsided. After one week of hospitalization, ceftriaxone was discontinued and the patient was discharged on doxycycline and rifampin.

Three sets of aerobic and anaerobic blood cultures were collected. After four days, all three aerobic blood culture vials were flagged positive-by Bactec FX^®^ system. Pleomorphic Gram-positive bacteria were isolated from the blood culture, ranging in shape from round cocci to short, thin rods bacilli Fig. (**1**). *Coryneform* species, usually interpreted as contaminants of unknown clinical importance, were suspected and a preliminary report of “Gram-positive bacilli” was generated and reported. After 24 hour- incubation on blood agar plates, tiny growth was observed on the primary streaking, further suggesting *Coryneform* isolates as many *Coryneform* species are characterized by slow growth. By 48 hrs, good growth was observed on a blood agar plate. Catalase test was positive, a characteristic commonly exhibited by both *Coryneform* species and *Brucella* species. *Coryneform* species are usually considered contaminants, however the presence of this organism in three consecutive vials prompted further identification by VITEK-2 Compact 60 (AES software) (bioMerieux, Inc., USA) automated system using the VITEK Anaerobe Identification (ANI) card. This was done to rule out *Corynebacterium urealyticum*, *C. jeikeium*, and *C. striatum*, which can be pathogenic in vulnerable individuals [13, 14]. After 18 hrs, the blood culture isolate remained unidentified on ANI by 2 different VITEK 2 system machines. Gram staining was repeated from the blood agar plate; pleomorphic Gram-variable bacteria were identified. The oxidase test using Remel BactiDrop™ Oxidase ampules (Thermo Scientific) was performed and found to be strongly positive, allowing *Coryneform* species to be excluded and *Brucella* to be suspected. The urease test was performed using Urea Agar slant tubes (Saudi Prepared Media Laboratory Co. Ltd) and found to be strongly positive after 3 hour- incubation, further suggesting *Brucella*.

PCR confirmation of *Brucella* identification was done by using real-time PCR method. Briefly, DNA was extracted using the MagNA-Pure-Compact-System^®^ (Roche Diagnostics, Mannheim, Germany), based on the manufacturer’s instructions. A total of 400 μl of blood was drawn from the blood culture positive bottle, poured into MagNa pure sample tube and DNA was extracted using the MagNA Pure LC DNA Isolation Kit I according to the manufacturer’s instructions. The DNA material was eluted in 50 μl total volume and *Brucella* detected by real-time PCR using the proprietary *Brucella* Real Time PCR Kit (Shanghai ZJ Bio-Tech Co., Ltd). The PCR thermal conditions were followed as described by the manufacturer. After the activation of the enzyme at 37°C for 2 min, 1 cycle and 94°C for 2 min, 1 cycle, and amplification of PCR target was done at 93°C for 5 sec for denaturation, 60°C for 30 sec for annealing for a total of 40 cycles. The amplification signal was measured at 60°C using 530 detection fluorescent channel in LightCycler 2.0 instrument.

#### Case 1 Recommendations and Discussion

Identification of pleomorphic Gram-positive bacilli after initial Gram staining from blood culture should be done by careful interpretation as both *Corynebacterium* and *Brucella* species have similar slow growth patterns. Not only the Gram staining result might be misinterpreted, but also the final identification; hence inappropriate treatment may be prescribed due to such misinterpretations. It is recommended that oxidase test from the plate and urease test from the culture vial be performed after apparent identification of pleomorphic Gram-positive bacilli, once tiny growth is observed, in order to distinguish *Brucella* from *Corynebacterium* species. *Brucella* infection is of particular concern in Saudi Arabia, given the high volume of livestock importation [11, 12]. Misidentification due to misleading initial Gram-staining results can also lead to inadvertent exposure of laboratory staff to *Brucella* [10]. Earlier detection would be helpful in ensuring subsequent appropriate handling of the relevant samples in the laboratory for example, in procedures that can result in aerosolization and protect other laboratory staff from exposure [10].

### Case Report 2

A 79-year-old Saudi man was admitted to an outside hospital on March 8, 2015, with pneumonia requiring intubation and mechanical ventilation. His medical history was significant for diabetes mellitus, hypertension, dyslipidemia, chronic renal insufficiency and ischemic heart disease. His hospital course was complicated by septic shock, acute renal failure on top of chronic renal insufficiency requiring hemodialysis. The patient was treated extensively with broad spectrum antibiotics.

The patient was transferred to Johns Hopkins Aramco Healthcare on March 30, 2015, and required mechanical ventilation. He was treated with imipenem and levofloxacin. The patient was extubated two days later and had to be re-intubated because of extensive respiratory secretions. Multiple blood cultures identified MDR Acinetobacter. He was treated successfully with colistin.

Three sets of aerobic and anaerobic blood cultures were collected. Sixteen hours later, two aerobic blood culture vials were flagged positive by the Bactec FX^®^ system. Preliminary Gram stain results indicated giant Gram-positive oval cocci from both vials, suggesting *Staphylococcus* species. A preliminary report of “Gram-positive bacilli” was generated and reported to the charge nurse of the floor where the patient was admitted. However, after 18 hrs, 1-2 mm non-pigmented, domed, and mucoid colonies with smooth surfaces were observed on blood agar and MacConkey agar, suggesting the presence of Gram-negative bacilli Fig. (**2**). Gram staining was repeated from both the plate and the vials, and the presence of Gram-negative rod-shaped organisms and giant Gram-positive oval shaped organisms was confirmed. The growth on the blood agar plate was confirmed as *A. baumanii* using the VITEK-2 Gram-Negative Identification test (GNI) card (bioMérieux. Marcy l’Etoile, France). DNA was extracted as described above and real-time PCR was carried as described previously. The PCR was carried out directly from the vial and the presence of *A. baumanii* was confirmed. A correction report was generated. A subculture of the blood culture was done on Columbia agar (Gram-positive media), blood agar, and MacConkey agar. 24 hours later, no growth was observed on Colombia media, while excellent growth was observed on blood agar and MacConkey agar, suggesting the absence of Gram-positive organisms. Vitek MS is also considered an excellent tool to identify the organisms directly from the blood culture vial regardless of their gram stain characteristics and their morphology . Thereby, it could be an excellent choice for gram variable organisms.

#### Case 2 Recommendations and Discussion

In logarithmic phase, *Acinetobacter* generally have the form of short Gram-negative rods, but usually become more coccoid in the stationary phase. As they are difficult to destain, they may be misidentified as either Gram-variable or Gram-positive cocci [1-3]. In this case, preliminary Gram-staining was misinterpreted and an identification of giant Gram-positive oval cocci was erroneously made. It is recommended that once giant Gram-positive oval cocci are visualized on preliminary Gram-staining from blood culture vials, the technologist should repeat the Gram stain from the plate after 4-6 h, or add a few drops of the cultured material into Triple Sugar Iron (TSI) medium and repeat the Gram stain after 2-4 h incubation at 37^°^C. Organisms including *Acinetobacter* do not ferment glucose, and are strictly aerobic, therefore they will not change the color of the TSI medium, unlike, for example, *Staphylococcus* which carries out acidic fermentation.

MDR- *Acinetobacter* is on the increase worldwide. There are few reports on the MDR properties of *A. baumannii* in Saudi Arabia, with evidence pointing to increases in nosocomial MDR- *A. baumannii* infections. Misinterpretations of initial Gram-staining results, such as occurred in case 2, could be avoided by applying the recommendations above. This would in turn help expedite antibiotic susceptibility testing on the *A. baumannii* isolates and hence identification of the most appropriate antibiotic treatment [15-18]. This recommended procedure would have many benefits for both patients and healthcare providers, including reductions in morbidity and mortality, fewer laboratory tests and procedures, fewer and shorter hospital and intensive care unit stays, and decreased financial costs [15-20].

## Figures and Tables

**Fig. (1):**
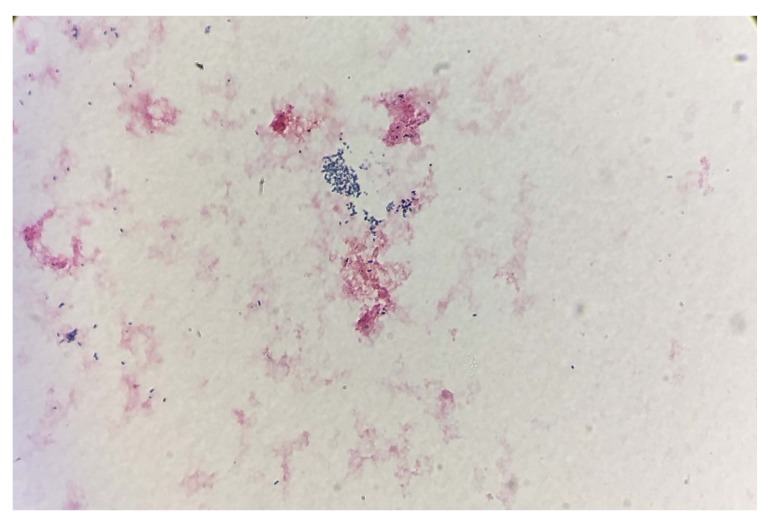
*Brucella* species staining gram-positive from a positive blood culture (× 1000).

**Fig. (2):**
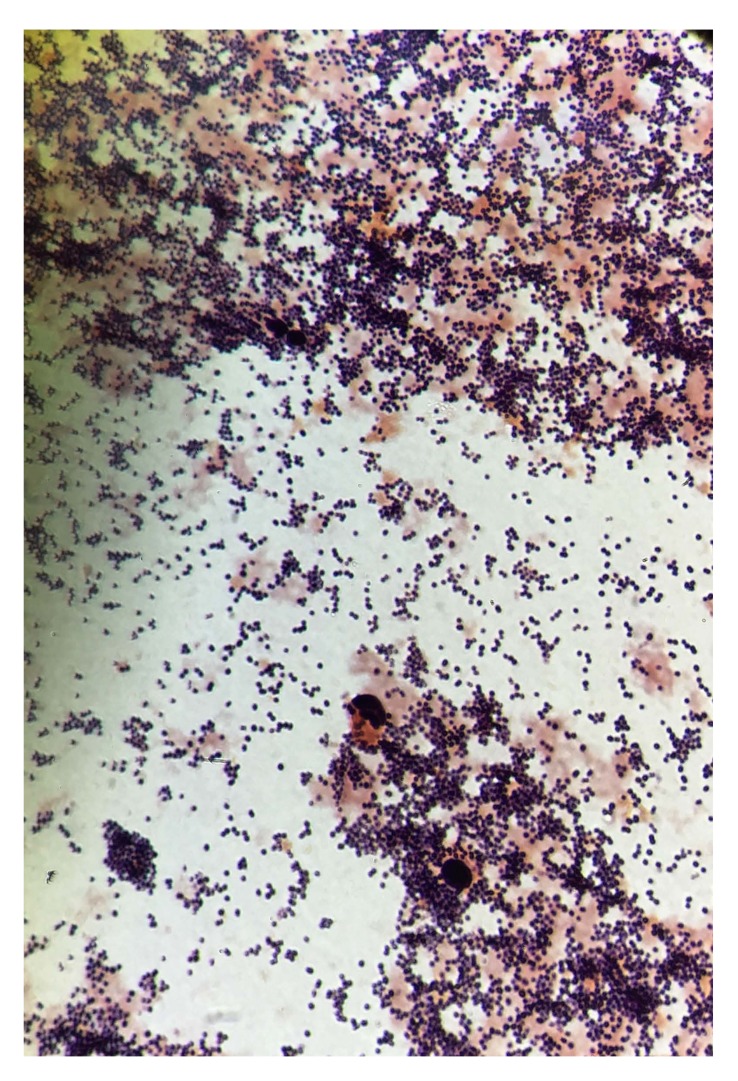
*Acinetobacter* staining gram-positive from a positive blood culture (× 1, 000).
